# Efficient Bounding Schemes for the Two-Center Hybrid Flow Shop Scheduling Problem with Removal Times

**DOI:** 10.1155/2014/605198

**Published:** 2014-12-21

**Authors:** Lotfi Hidri, Anis Gharbi, Mohamed Aly Louly

**Affiliations:** Industrial Engineering Department, College of Engineering, King Saud University, P.O. Box 800, Riyadh 11421, Saudi Arabia

## Abstract

We focus on the two-center hybrid flow shop scheduling problem with identical parallel machines and removal times. The job removal time is the required duration to remove it from a machine after its processing. The objective is to minimize the maximum completion time (makespan). A heuristic and a lower bound are proposed for this NP-Hard problem. These procedures are based on the optimal solution of the parallel machine scheduling problem with release dates and delivery times. The heuristic is composed of two phases. The first one is a constructive phase in which an initial feasible solution is provided, while the second phase is an improvement one. Intensive computational experiments have been conducted to confirm the good performance of the proposed procedures.

## 1. Introduction

The hybrid flow shop (HFS) manufacturing systems are frequently encountered in the electronic industry, such as in PCB fabrication and integrated circuit (IC) packaging. HFS is composed of a series of production centers. Each one of these production centers includes several parallel machines. Some centers may contain only one machine but at least one center contains more than one machine. These parallel machines (for each center) are intended to treat several jobs simultaneously and may be identical, unrelated, or uniform.

The jobs have to follow the same route from the first center to the last one during the processing (this is the flow side of the HFS). Each job can be treated by any one of the machines included in a center but must be processed by only one machine. Each machine can process one job at the same time without preemption for a fixed amount of time. In addition, the buffer capacity between the consecutive centers is assumed to be unlimited. The main purpose is to find a feasible schedule that minimizes a given criterion such as the maximum completion time or the mean flow time.

During the modeling phase, several practical assumptions are neglected in order to simplify the mathematical treatment of the corresponding scheduling problem. Indeed, despite its practical importance in some manufacturing systems, the removal time is commonly either ignored or considered as a hidden part of the processing time. Thus taking into account the removal time will reduce the gap between theory and practice.

The removal time for a job is the required time to remove it from a given machine after being processed. The removal of the treated jobs from machines may be a time consuming procedure; thus accurate models have to consider the removal times. The removal time is of two types:

- sequence-independent removal time: the removal time depends only on the considered job;
- sequence-dependent removal time: the removal time depends on the considered job and its predecessor.

The scheduling problems with removal times are in general NP-Hard even for a small system [1]. While checking the literature on the scheduling problems with removal times, only few papers have been found; among them we quote [2–9].

Hybrid flow shop scheduling problem with removal times (HFSRT) is further challenging because it is more complex than the small systems. More precisely, the HFSRT is strongly NP-Hard, since a particular case which is the two-center hybrid flow shop scheduling problem is strongly NP-Hard [10]. Moreover, HFSRT is of a practical interest since it models several real life situations in manufacturing. Surprisingly, the literature for HFSRT is scant and only a few papers on the subject were provided. In this context, the authors in [11] addressed the two-center hybrid flow shop problem with removal and setup times and they developed several heuristics to minimize the makespan. In [12], the hybrid flow shop scheduling problem with setup times, removal times, due dates, and precedence constraints is addressed and six heuristics are developed to minimize the maximum lateness.

In this paper, we focus on the two-center hybrid flow shop scheduling problem with sequence-independent removal times. We develop a new two-phase heuristic procedure, which utilizes iteratively the optimal solution of the parallel machine scheduling problem. In addition, we propose a lower bound which is based on relaxing the two-center hybrid flow shop scheduling problem with sequence-independent removal times into two parallel machine scheduling problems. These two parallel machine scheduling problems are solved optimally. The developed lower bound is intended to evaluate the performance of the proposed heuristic.

The rest of this paper is organized as follows. In Section 2, a formal definition of the studied scheduling problem is given, along with some useful properties.  Section 3 introduces the parallel machine scheduling problem. A lower bounding scheme is presented in Section 4. The developed heuristic algorithm is detailed in Section 5.  Section 6 is devoted to test the performance of the proposed procedures. Finally, some conclusions and future directions are presented in Section 7.

## 2. Problem Description

In this paper we address the two-center hybrid flow shop scheduling problem with identical parallel machines and removal times (HFSRT). The HFSRT is stated as follows. We are given a set *J* = {1,2,…, *n*} of *n* jobs and two centers *C*_1_ and *C*_2_. Each center *C*_*i*_ contains *m*_*i*_  (*i* = 1,2) identical parallel machines, *M*_*i*,1_, *M*_*i*,2_,…, *M*_*i*,*m*_*i*__. Each job *j* ∈ *J* has to be processed first on a machine of the first center *C*_1_, without preemption, during *p*_1,*j*_ units of time. Once the processing of the job *j* is completed, a removal time *rm*_1,*j*_ is required to remove the job *j* from the machine on which it has been treated. After that, the job *j* is transferred to the second center *C*_2_ where it will be processed on an available machine, without preemption, for *p*_2,*j*_ units of time. After that its removal will last *rm*_2,*j*_ units of time. The objective is to find a feasible schedule that minimizes the last completion time or the makespan *C*_max⁡_.

All jobs and all machines are available from time zero. All the processing times *p*_*ij*_ and the removal times *rm*_*i*,*j*_ (*i* = 1,2 and *j* ∈ *J*) are integer and deterministic. In addition, the intermediate storage between the two centers is assumed to be unlimited. Following the three-field notation *α* | *β* | *γ*, the HFSRT problem is noted *F*_2_(*P*_*m*_1__, *P*_*m*_2__)|*rm*_*ij*_|*C*_max⁡_ [13]. In the following, we define the modified processing time of the job *j* ∈ *J* in center *C*_*i*_  (*i* = 1,2) as ${\overset{-}{p}}_{ij}=p_{ij}+rm_{i,j}$.

**Example 1 .**

We consider the following instance: *m*_1_ = *m*_2_ = 2 and *n* = 5. The processing times of Example and the removal times are displayed as follows:

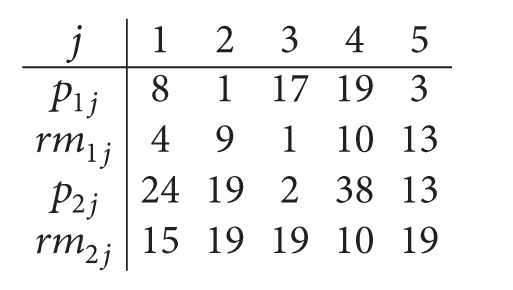
(1)

A feasible schedule for Example 1 with maximum completion time *C*_max⁡_ = 125 is given by

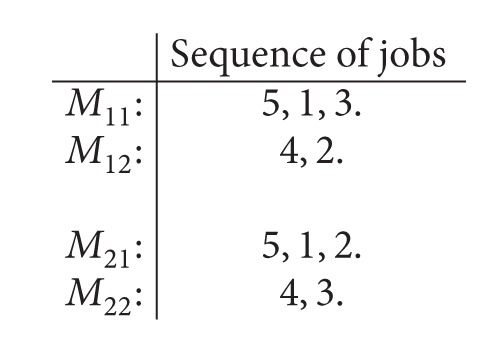
(2)


In order to enhance the value of the makespan we introduce the reverse problem of *F*_2_(*P*_*m*_1__, *P*_*m*_2__)|*rm*_*ij*_|*C*_max⁡_ which is the problem obtained by inverting the roles of the centers in addition to the permutation of the processing and removal times. More precisely, if *C*_*i*_^*R*^  (*i* = 1,2) denotes the *i*th center for the reverse problem, then we have *C*_1_^*R*^ = *C*_2_ and *C*_2_^*R*^ = *C*_1_. In addition, if *p*_*ij*_^*R*^, *rm*_*i*,*j*_^*R*^  (*i* = 1,2 and *j* ∈ *J*) are the processing times and removal times for the reverse problem, respectively, then *p*_1*j*_^*R*^ = *rm*_2,*j*_, *rm*_1,*j*_^*R*^ = *p*_2*j*_,  *p*_2*j*_^*R*^ = *rm*_1,*j*_, and *rm*_2,*j*_^*R*^ = *p*_1*j*_  (*j* ∈ *J*). The reverse problem is an interesting one since it has the same optimal makespan as for the original problem. This claim can be proved by considering the linear transformation *t* ^*R*^ = *C*_max⁡_ − *t*, where *t* is the time in the original problem and *t* ^*R*^ is the time in the reverse problem.

## 3. The Parallel Machine Scheduling Problem with Release Dates and Delivery Times

The proposed upper and lower bounding procedures are based on the optimal solution of the parallel machine scheduling problem with release dates and delivery times, which is defined as follows. A set *J* = {1,2,…, *n*} of *n* jobs has to be processed on *m* parallel machines *M*_1_, *M*_2_,…, *M*_*m*_ without preemption. Each machine can handle one job at most at the same time and each job *j* ∈ *J* is characterized by the following:

- *r*_*j*_: a release date from which the job *j* is ready to be treated;
- *p*_*j*_: a processing time;
- *q*_*j*_: a delivery time which is the minimum elapsed time between the completion of the job *j* and exiting the system.

If *t*_*j*_ is the starting time of the job *j* in a feasible schedule then its completion time is *c*_*j*_ = *t*_*j*_ + *p*_*j*_ + *q*_*j*_ and the makespan is *C*_max⁡_ = max⁡_*j*∈*J*_⁡(*c*_*j*_). The objective is to find a feasible schedule that minimizes the makespan. This problem is denoted by *P*_*m*_|*r*_*j*_, *q*_*j*_|*C*_max⁡_. This problem is well studied in the literature and a plenty of papers have been provided. The *P*_*m*_|*r*_*j*_, *q*_*j*_|*C*_max⁡_ is strongly NP-Hard [14]. In order to solve this problem, several exact methods have been proposed and the most efficient method is presented in [15], which will be used in this work. It is worth noting that this exact algorithm is based on a Branch and Bound procedure, where a fixed time limit is set to solve the *P*_*m*_|*r*_*j*_, *q*_*j*_|*C*_max⁡_ problem. If the exact algorithm fails to reach an optimal solution for the *P*_*m*_|*r*_*j*_, *q*_*j*_|*C*_max⁡_ problem within this time limit, the best lower and upper bounds are retrieved. Interestingly, the *P*_*m*_|*r*_*j*_, *q*_*j*_|*C*_max⁡_ problem is equivalent to the the parallel machine scheduling problem with release dates and maximum lateness: *P*_*m*_|*r*_*j*_, *d*_*j*_|*L*_max⁡_, where

- *r*_*j*_: a release date from which the job *j* is ready to be treated;
- *d*_*j*_: the due date;
- *L*_max⁡_ = max⁡_*j*∈*J*_⁡*L*_*j*_ and *L*_*j*_ = *t*_*j*_ + *p*_*j*_ − *d*_*j*_, *t*_*j*_ being the starting time of the job *j*.

The *P*_*m*_|*r*_*j*_, *d*_*j*_|*L*_max⁡_ problem is required for some steps in the development of the heuristic procedure for the two-center hybrid flow shop scheduling problem with identical parallel machines and removal times.

## 4. A Lower Bounding Procedure

In this section, we present a lower bound for the HFSRT. For that aim, we define for each job *j* ∈ *J* and for each center *C*_*i*_(*i* = 1,2) a release date *r*_*ij*_ and a delivery time *q*_*ij*_ that are expressed as follows:

(3) rij=0 if  i=1,rij=p−1j if  i=2,qij=p−2j if  i=1,qij=0 if  i=2.

(i) By relaxing the capacity of the second center *C*_2_ (i.e., the number of machines *m*_2_ is assumed to be infinite), we obtain in the first center *C*_1_ a parallel machine scheduling problem with release dates and delivery times *P*_*m*_|*r*_*j*_, *q*_*j*_|*C*_max⁡_. The data is as follows:
  (4) $$\begin{array}{c} m=m_{1},\\ r_{j}=r_{1j}=0,\\ p_{j}={\overset{-}{p}}_{1j},\\ q_{j}=q_{1j}={\overset{-}{p}}_{2j}. \end{array}$$

The optimal value while solving the corresponding *P*_*m*_|*r*_*j*_, *q*_*j*_|*C*_max⁡_ problem provides the first lower bound *LB*_1_.

(ii) Similarly, by relaxing the capacity of the first center *C*_1_ (i.e., the number of machines *m*_1_ is assumed to be infinite), we obtain in the second center *C*_2_ a parallel machine scheduling problem with release date and delivery time *P*_*m*_|*r*_*j*_, *q*_*j*_|*C*_max⁡_. The data is as follows:
  (5) $$\begin{array}{c} m=m_{2},\\ r_{j}=r_{2j}={\overset{-}{p}}_{1j},\\ p_{j}={\overset{-}{p}}_{2j},\\ q_{j}=q_{2j}=0. \end{array}$$

The optimal value while solving the corresponding *P*_*m*_|*r*_*j*_, *q*_*j*_|*C*_max⁡_ problem provides the second lower bound LB_2_.

Obviously, a valid lower bound for the HFSRT is

(6) $$\begin{array}{c} \text{LB}=\max\left({\text{LB}}_{1},{\text{LB}}_{2}\right). \end{array}$$

In order to illustrate the computation of the lower bound LB, we reconsider the data given in Example 1.

(iii) By relaxing the capacity of the second center, we obtain a parallel machine scheduling problem *P*_*m*_|*r*_*j*_, *q*_*j*_|*C*_max⁡_ in the first center with *m* = *m*_1_ = 2. Data after relaxing the capacity of *C*_2_ is

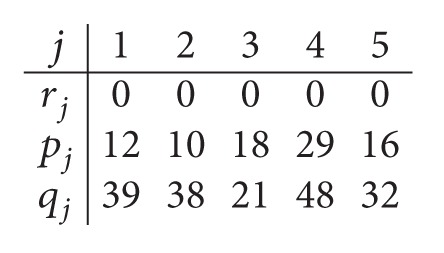
(7)
 The optimal schedule is given by the sequence (4,3) on machine *M*_11_ and the sequence (1,2, 5) on machine *M*_12_. The optimal solution value is *C*_max⁡_^*^ = 77. Consequently, *LB*_1_ = 77.
(iv) Similarly, the relaxation of the capacity of the first center yields a parallel scheduling problem in the second center with *m* = *m*_1_ = 2. Data after relaxing the capacity of *C*_1_is

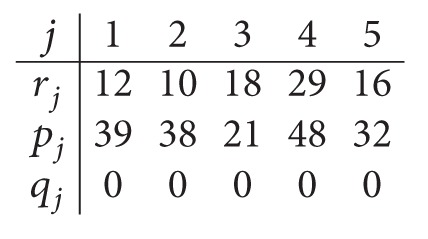
(8)

The corresponding optimal schedule is the sequence (4,1) on machine *M*_21_ and the sequence (3,5, 2) on machine *M*_22_.

The optimal solution *C*_max⁡_^*^ = 101; thus LB_2_ = 101. Consequently, LB = max⁡(LB_1_, LB_2_) = 101.

## 5. A Heuristic Approach

This section is devoted to the presentation of the developed heuristic for the HFSRT. This heuristic is a two-phase procedure. The first one is constructive phase in which an initial feasible solution is provided. The second phase is an improvement phase. The core of this heuristic is the utilization of the optimal solution of the parallel machine scheduling problem with release dates and delivery times *P*_*m*_|*r*_*j*_, *q*_*j*_|*C*_max⁡_ and its equivalent version, which is the parallel machine scheduling problem with release dates and maximum lateness criterion *P*_*m*_|*r*_*j*_, *d*_*j*_|*L*_max⁡_. The two phases of the heuristic are detailed in the following.

**Phase 1 (initial feasible solution).**

* *

*Step  1.1*. For each job *j* ∈ *J*, set *r*_*j*_ = 0, $p_{j}={\overset{-}{p}}_{1j}$,  $q_{j}={\overset{-}{p}}_{2j}$, and the number of machines *m* = *m*_1_.

*Step  1.2*. Solve optimally the *P*_*m*_|*r*_*j*_, *q*_*j*_|*C*_max⁡_ problem, with the given data in Step 1.1. Denote by *c*_1**j**_ the obtained completion time for *j* ∈ *J*.

*Step  1.3*. For each job *j* ∈ *J*, set *r*_*j*_ = *c*_1**j**_, $p_{j}={\overset{-}{p}}_{2j}$, *q*_*j*_ = 0, and the number of machines *m* = *m*_2_.

*Step  1.4*. Solve optimally the *P*_*m*_|*r*_*j*_, *q*_*j*_|*C*_max⁡_ problem, with the given data in Step 1.3. Denote by *t*_2**j**_ the obtained starting time of job *j* ∈ *J*.

*Step  1.5*. Set $UB=\max_{j\in J}\{t_{2j}+{\overset{-}{p}}_{2j}\}$.

**Phase 2 (improvement phase).**

* *

*Step  2.1*. For each job *j* ∈ *J*, set *r*_*j*_ = 0,  $p_{j}={\overset{-}{p}}_{1j}$, *d*_*j*_ = *t*_2*j*_, and the number of machines *m* = *m*_1_.

*Step  2.2*. Solve optimally the *P*_*m*_|*r*_*j*_|*L*_max⁡_ problem, with the given data in Step 2.1. Denote by *c*_1**j**_ the obtained completion time for *j* ∈ *J*. If *L*_max⁡_ = 0 then* STOP*, or else set *UB* : = *UB* + *L*_max⁡_.

*Step  2.3*. For each job *j* ∈ *J*, set *r*_*j*_ = *c*_1**j**_, $p_{j}={\overset{-}{p}}_{2j}$, *q*_*j*_ = 0, and the number of machines *m* = *m*_2_.

*Step  2.4*. Solve optimally the *P*_*m*_|*r*_*j*_, *q*_*j*_|*C*_max⁡_ problem, with the given data in Step 2.3. Denote by *t*_2*j*_ the obtained starting time for *j* ∈ *J*. If *C*_max⁡_ < *UB* then set *UB* : = *C*_max⁡_.

*Step  2.5*. Go to Step 2.1.

In the first phase, Step 1.1 and Step 1.2 provide a feasible schedule on the first center. During Step 1.3 and Step 1.4 a feasible schedule in the second center is constructed. The latter schedule does not contradict the first one since the release date in the second center is the completion time in the first center for each job. Thus, a concatenation of the two schedules is possible. This allows to get of a feasible schedule for the HFSRT with makespan *UB*.

In the second phase, we first fix the schedule in the second center and we try to reschedule the jobs in the first center in order to reduce the makespan. Since we have set the due dates *d*_*j*_ in the first center as the starting time *t*_2**j**_ on the second center, we ensure the existence of a feasible schedule (the existing one) in the first center and consequently *L*_max⁡_ ≤ 0. If *L*_max⁡_ < 0 then the obtained schedule in the first center allows the improvement of the makespan by *UB* + *L*_max⁡_. Now, we fix the schedule in the first center and we try to reschedule the jobs in the second center. If the obtained schedule has a makespan *C*_max⁡_ < *UB*, then there is an improvement and *UB* : = *C*_max⁡_. All the previous operations are repeated until no improvement is reached. In the sequel we illustrate all the steps of the heuristic on Example 1.

**Example 1 (continued).**

* *

*Step  1.1*. The data for the parallel machine scheduling problem in the first center are given as follows:

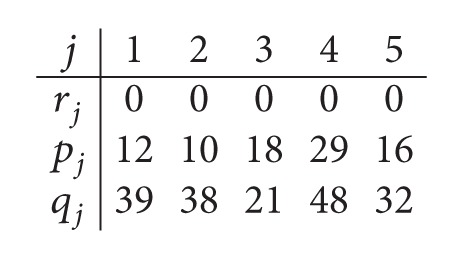
(9)

*Step  1.2*. After solving optimally the *P*_*m*_|*r*_*j*_, *q*_*j*_|*C*_max⁡_ problem, with the given data in Step 1.1, the obtained completion times after Step 1.1 are given as follows:

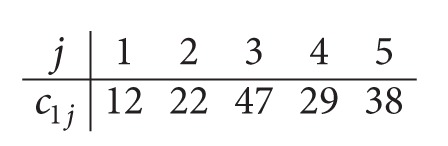
(10)

*Step  1.3*. Thus, the data for the parallel machine scheduling problem in the second center are given as follows:

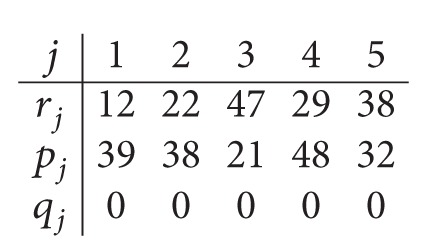
(11)

*Step  1.4*. After solving the *P*_*m*_|*r*_*j*_, *q*_*j*_|*C*_max⁡_ problem, we obtain the optimal schedule with the sequence (1,3, 5) on machine *M*_21_ and the sequence (2,4) on machine *M*_22_. The optimal value is *C*_max⁡_^*^ = *UB* = 108 and the starting times *t*_2**j**_ are given as follows:

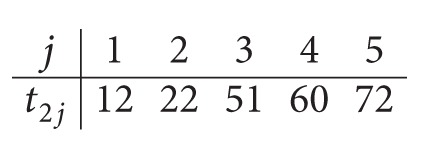
(12)

*Step  1.5*. The obtained maximum completion time is *UB* = 108.

*Step  2.1*. The data for *P*_*m*_|*r*_*j*_|*L*_max⁡_ are given as follows:

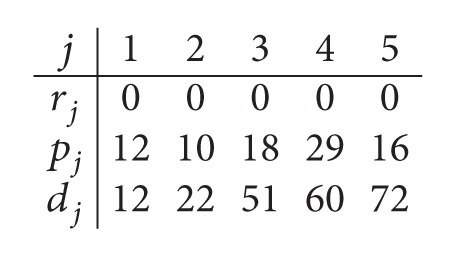
(13)

*Step  2.2*. After solving the *P*_*m*_|*r*_*j*_|*L*_max⁡_ problem, we get the sequence (2,3, 5) on *M*_21_ and the sequence (1,4) on machine *M*_22_. The optimal value is *L*_max⁡_^*^ = −4. Thus, the makespan is improved and *UB* = *UB* + *L*_max⁡_^*^ = 104. The completion times are given as follows:

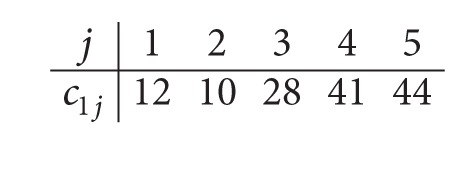
(14)

*Step  2.3*. For this step the data is given as follows:

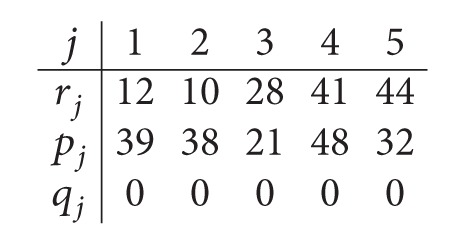
(15)

The optimal makespan is *UB* = 101. Therefore, we have *UB* = LB = 101 and an optimal solution is reached. The optimal sequences are (2,3, 5) on *M*_11_ and *M*_21_ and (1,4) on *M*_12_ and *M*_22_.

It is worth noting that the heuristic is systematically applied to the reverse problem in order to improve the obtained value of the makespan. For instance, investigating the reverse problem of Example 1 will provide *UB* ^*R*^ = 101 in the first phase of the heuristic.

## 6. Numerical Experiments

In the sequel, we analyze the empirical performance of the developed procedures. These procedures are coded in C and implemented on a Pentium dual core, 1.7 GHz Personal Computer with 504 Mo RAM.

### 6.1. Test Problems

The instances are generated as for [16].

- The number of jobs *n* ∈ {10,20,50,100,200}.
- The number of machines (*m*_1_, *m*_2_) ∈ {(2,2), (2,4), (4,2), (4,4)}.
- The processing times and the removal times *p*_1,*j*_, *p*_2,*j*_, *rm*_1,*j*_, and *rm*_2,*j*_  (*j* ∈ *J*) are generated as follows:
  - *p*_1,*j*_ is generated uniformly from [1, *a*],
  - *rm*_1,*j*_ is generated uniformly from [1, *b*],
  - *p*_2,*j*_ is generated uniformly from [1, *c*],
  - *rm*_2,*j*_ is generated uniformly from [1, *d*].

With *a* = *c* = 20 and *b*, *d* ∈ {20,40}, for each combination *n*, *m*_1_, *m*_2_, *a*, *b*, *c*, and *d*, 10 instances are generated which result in 800 instances.

### 6.2. Performance Analysis

The relative gap which is defined by *G* = 100 × ((*UB* − LB)/LB) is intended to assess the performance of the proposed heuristic. According to the experimental results, we observe that the proposed procedures are very effective since the mean relative gap is 0.506% and the percentage of the optimally solved instances (i.e., *UB* = LB) is 77.5%. In addition, the mean required time for providing a feasible solution is 7.105 seconds. A more detailed analysis for each combination (80 combinations with 10 instances per combination) is presented in Tables 1, 2, 3, and 4, where

- %*S*: percent of solved instances;
- MT: mean required time for solving an instance (in seconds);
- MG: the mean relative gap;
- *Max*⁡*G*: the maximum relative gap.

Based on Tables 1, 2, 3, and 4 we observe that the proposed procedures are able to solve large instances (up to 200 jobs) within moderate time. In addition, the instances with balanced workload in the two centers ((*a* + *b*)/*m*_1_ = (*c* + *d*)/*m*_2_) are the most hard instances to be solved. Furthermore, the increasing of the number of machines makes also instances hard to solve. If the workload in the two centers is not balanced ((*a* + *b*)/*m*_1_ ≠ (*c* + *d*)/*m*_2_), most of the instances are solved. We observe also that the balanced small sized instances (10 and 20 jobs) are harder to be solved than the balanced large sized ones.

## 7. Conclusion

In this paper, we proposed a lower bound and a heuristic for the two-center hybrid flow shop scheduling problem with identical parallel machines and removal times. The scope of this paper is the consideration of the removal times for such a scheduling problem. The proposed bounding schemes are based on the optimal solution of the parallel machine scheduling problem with release dates and delivery times. The lower bound is intended to evaluate the performance of the heuristic through the relative gap. The proposed heuristic is a two-phase algorithm, where the first phase is a constructive one and the second phase is an improvement phase. Intensive experimental computations are conducted and the results demonstrate the efficiency of the proposed procedures, since the mean relative gap is about 0.506%.

Future research is required for the *F*_2_(*P*_*m*_1__, *P*_*m*_2__)|*rm*_*ij*_|*C*_max⁡_ scheduling problem, especially in terms of the development of exact solutions. In addition, other variants of the current studied scheduling problem have to be investigated such as the consideration of the learning effect. More attention must be paid for the balanced small sized instances by proposing, for example, linear programming models.

## Figures and Tables

**Table 1:** Performance for *a* = *b* = *c* = *d* = 20.

*n*	*m*_1_	*m*_2_	%*S*	MT (s)	MG	Max*G*
10	2	2	40	0.058	1.988	6.977
10	2	4	100	0.011	0.000	0.000
10	4	2	90	0.030	0.455	4.545
10	4	4	60	0.030	2.262	7.229
20	2	2	40	0.075	1.693	5.742
20	2	4	100	0.016	0.000	0.000
20	4	2	100	4.924	0.000	0.000
20	4	4	10	1.458	5.531	15.000
50	2	2	60	10.120	1.032	5.351
50	2	4	100	0.047	0.000	0.000
50	4	2	100	3.453	0.000	0.000
50	4	4	50	13.014	1.192	3.676
100	2	2	40	30.514	0.311	0.898
100	2	4	100	0.183	0.000	0.000
100	4	2	90	1.697	0.010	0.097
100	4	4	50	5.355	0.726	3.429
200	2	2	90	32.933	0.034	0.344
200	2	4	80	2.250	0.010	0.049
200	4	2	80	6.156	0.014	0.093
200	4	4	60	20.451	0.370	1.507

**Table 2:** Performance for *a* = *b* = *c* = 20 and *d* = 40.

*n*	*m*_1_	*m*_2_	%*S*	MT (s)	MG	Max*G*
10	2	2	70	0.178	0.497	2.158
10	2	4	70	0.008	0.959	5.785
10	4	2	100	0.256	0.000	0.000
10	4	4	50	0.025	1.779	8.642
20	2	2	90	2.042	0.159	1.587
20	2	4	100	0.230	0.000	0.000
20	4	2	100	2.598	0.000	0.000
20	4	4	80	6.378	0.311	2.548
50	2	2	70	0.128	0.050	0.247
50	2	4	80	0.138	0.140	0.840
50	4	2	100	0.783	0.000	0.000
50	4	4	90	16.527	0.026	0.264
100	2	2	70	10.617	0.051	0.385
100	2	4	90	0.772	0.062	0.618
100	4	2	100	1.449	0.000	0.000
100	4	4	80	25.697	0.027	0.135
200	2	2	90	4.339	0.006	0.064
200	2	4	100	2.058	0.000	0.000
200	4	2	100	7.931	0.000	0.000
200	4	4	70	6.305	0.026	0.133

**Table 3:** Performance for *a* = *c* = *d* = 20 and *b* = 40.

*n*	*m*_1_	*m*_2_	%*S*	MT (s)	MG	Max*G*
10	2	2	60	0.100	0.812	3.185
10	2	4	100	0.230	0.000	0.000
10	4	2	90	0.052	0.087	0.870
10	4	4	60	0.020	1.450	7.317
20	2	2	70	10.016	0.356	1.608
20	2	4	100	0.008	0.000	0.000
20	4	2	50	23.989	0.399	2.155
20	4	4	90	6.234	0.357	3.571
50	2	2	80	0.059	0.051	0.385
50	2	4	100	0.042	0.000	0.000
50	4	2	70	12.025	0.133	0.575
50	4	4	80	48.123	0.053	0.270
100	2	2	100	0.266	0.000	0.000
100	2	4	100	0.520	0.000	0.000
100	4	2	90	9.955	0.010	0.099
100	4	4	80	25.181	0.054	0.409
200	2	2	90	12.427	0.007	0.065
200	2	4	100	2.447	0.000	0.000
200	4	2	90	11.502	0.005	0.049
200	4	4	70	8.006	0.039	0.195

**Table 4:** Performance for *a* = *c* = 20 and *b* = *d* = 40.

*n*	*m*_1_	*m*_2_	%*S*	MT (s)	MG	Max*G*
10	2	2	50	0.097	3.812	10.417
10	2	4	100	0.109	0.000	0.000
10	4	2	100	0.155	0.000	0.000
10	4	4	10	0.028	5.153	11.628
20	2	2	30	1.725	1.679	5.519
20	2	4	100	0.072	0.000	0.000
20	4	2	100	0.107	0.000	0.000
20	4	4	10	17.413	2.381	7.222
50	2	2	60	0.177	0.449	1.906
50	2	4	100	0.078	0.000	0.000
50	4	2	100	6.909	0.000	0.000
50	4	4	20	21.588	1.705	5.600
100	2	2	70	14.164	0.286	1.613
100	2	4	100	0.373	0.000	0.000
100	4	2	100	3.602	0.000	0.000
100	4	4	40	20.481	0.825	2.857
200	2	2	70	13.336	0.154	0.790
200	2	4	90	2.313	0.010	0.099
200	4	2	80	27.520	0.016	0.127
200	4	4	60	45.717	0.464	1.570
